# A phase 1/2, open‐label, multicenter study of isatuximab in combination with cemiplimab in patients with lymphoma

**DOI:** 10.1002/hon.3089

**Published:** 2022-10-31

**Authors:** Carmelo Carlo‐Stella, Pier Luigi Zinzani, Anna Sureda, Luis Araújo, Olivier Casasnovas, Cecilia Carpio, Su‐Peng Yeh, Krimo Bouabdallah, Guillaume Cartron, Won Seog Kim, Raul Cordoba, Youngil Koh, Alessandro Re, Daniela Alves, Martine Chamuleau, Steven Le Gouill, Armando López‐Guillermo, Ilídia Moreira, Marjolein W. M. van der Poel, Giovanni Abbadessa, Robin Meng, Ran Ji, Lucie Lépine, Rao Saleem, Vincent Ribrag

**Affiliations:** ^1^ Department of Biomedical Sciences Humanitas University and Department of Oncology and Hematology IRCCS Humanitas Research Hospital Milano Italy; ^2^ IRCCS Azienda Ospedaliero‐Universitaria di Bologna Istituto di Ematologia “Seràgnoli” and Dipartimento di Medicina Specialistica Diagnostica e Sperimentale Università di Bologna Bologna Italy; ^3^ Institut Català D'Oncologia ‐ Hospital Duran i Reynals IDIBELL Universitat de Barcelona Barcelona Spain; ^4^ Universitário de Coimbra Coimbra Portugal; ^5^ Hématologie Clinique, CHU Dijon Bourgogne Dijon France; ^6^ Department of Hematology Vall d'Hebron Institute of Oncology (VHIO) Hospital Universitari Vall d’Hebron Vall d’Hebron Barcelona Hospital Campus Universitat Autònoma de Barcelona Barcelona Spain; ^7^ China Medical University Hospital Taichung Taiwan; ^8^ Hematology and Cellular Therapy Department University Hospital of Bordeaux Bordeaux France; ^9^ Department of Hematology Centre Hospitalier Universitaire Montpellier Montpellier France; ^10^ Sungkyunkwan University School of Medicine Samsung Medical Center Seoul Korea; ^11^ Fundación Jiménez Díaz University Hospital Madrid Spain; ^12^ Department of Internal Medicine Seoul National University Hospital Seoul Korea; ^13^ Hematology Division ASST Spedali Civili Brescia Brescia Italy; ^14^ Hematology and Bone Marrow Transplant Department Hospital de Santa Maria Centro Hospitalar Universitário Lisboa Norte (CHULN) Lisbon Portugal; ^15^ Department of Hematology Cancer Center Amsterdam on behalf of the LLPC (Lunenburg Lymphoma Phase I/II Consortium) Amsterdam University Medical Center VU University Amsterdam Amsterdam The Netherlands; ^16^ Department of Hematology Institut Curie Paris France; ^17^ Department of Hematology Hospital Clínic de Barcelona Barcelona Spain; ^18^ Department of Onco‐Hematology Portuguese Institute of Oncology of Porto Porto Portugal; ^19^ Department of Internal Medicine Division of Hematology GROW School for Oncology and Developmental Biology on behalf of the LLPC (Lunenburg Lymphoma Phase I/II Consortium) Maastricht University Medical Center Maastricht the Netherlands; ^20^ Sanofi Cambridge Massachusetts USA; ^21^ Sanofi Chilly‐Mazarin France; ^22^ Département d’Hématologie et Département des Essais Précoces (DITEP) Institut Gustave Roussy Villejuif France

**Keywords:** cemiplimab, diffuse large B‐cell lymphoma, isatuximab, non‐Hodgkin lymphoma, peripheral T‐cell lymphoma

## Abstract

Patients with relapsed or refractory lymphoma have limited treatment options, requiring newer regimens. In this Phase 1/2 study (NCT03769181), we assessed the safety, efficacy, and pharmacokinetics of isatuximab (Isa, anti‐CD38 antibody) in combination with cemiplimab (Cemi, anti‐programmed death‐1 [PD‐1] receptor antibody; Isa + Cemi) in patients with classic Hodgkin lymphoma (cHL), diffuse large B‐cell lymphoma (DLBCL), and peripheral T‐cell lymphoma (PTCL). In Phase 1, we characterized the safety and tolerability of Isa + Cemi with planned dose de‐escalation to determine the recommended Phase 2 dose (RP2D). Six patients in each cohort were treated with a starting dose of Isa + Cemi to determine the RP2D. In Phase 2, the primary endpoints were complete response in Cohort A1 (cHL anti‐PD‐1/programmed death‐ligand 1 [PD‐L1] naïve), and objective response rate in Cohorts A2 (cHL anti‐PD‐1/PD‐L1 progressors), B (DLBCL), and C (PTCL). An interim analysis was performed when the first 18 (Cohort A1), 12 (Cohort A2), 17 (Cohort B), and 11 (Cohort C) patients in Phase 2 had been treated and followed up for 24 weeks. Isa + Cemi demonstrated a manageable safety profile with no new safety signals. No dose‐limiting toxicities were observed at the starting dose; thus, the starting dose of each drug was confirmed as the RP2D. Based on the Lugano 2014 criteria, 55.6% (Cohort A1), 33.3% (Cohort A2), 5.9% (Cohort B), and 9.1% (Cohort C) of patients achieved a complete or partial response. Pharmacokinetic analyses suggested no effect of Cemi on Isa exposure. Modest clinical efficacy was observed in patients with cHL regardless of prior anti‐PD‐1/PD‐L1 exposure. In DLBCL or PTCL cohorts, interim efficacy analysis results did not meet prespecified criteria to continue enrollment in Phase 2 Stage 2. Isa + Cemi did not have a synergistic effect in these patient populations.

## 1 INTRODUCTION

Immune checkpoint blockade has contributed to the efficacy of targeted anti‐programmed death 1 (PD‐1)/anti‐programmed death‐ligand 1 (PD‐L1) agents in many tumor types, with clinical responses observed in a small proportion of patients with Hodgkin lymphoma (HL) and rare non‐HL (NHL) subtypes.^1^, ^2^ PD‐1 inhibitors, nivolumab and pembrolizumab, received US Food and Drug Administration approval for relapsed/refractory classic Hodgkin lymphoma (cHL); however, only a small fraction of patients achieved complete response (CR) in Phase 2 trials of these agents.^3^, ^4^ Early‐phase studies with single‐agent anti‐PD‐1 antibodies produced low or modest clinical activity in patients with diffuse large B‐cell lymphoma (DLBCL) and peripheral T‐cell lymphoma (PTCL).^5^, ^6^, ^7^, ^8^ New therapies that employ anti‐PD‐1/PD‐L1 antibodies in combination with other therapies are being evaluated in patients with lymphoma.

The expression of the transmembrane glycoprotein CD38 is well documented in hematological cancers, including multiple myeloma (MM), certain types of lymphoma, and leukemia; the prevalence and expression level of CD38 is lower and more variable in non‐MM cancers.^2^, ^9^, ^10^, ^11^, ^12^, ^13^, ^14^ Monoclonal antibodies targeting CD38 have demonstrated deep clinical outcomes in patients with MM, but data supporting their clinical utility in lymphoid malignancies are limited.^15^ Preclinical studies demonstrated cytotoxic activity of anti‐CD38 antibodies against CD38+ malignancies.^9^, ^16^, ^17^ Furthermore, in murine models of lung cancer and melanoma, acquired resistance to anti‐PD‐1/PD‐L1 agents is associated with CD38 upregulation on tumor cells, thereby leading to CD38‐mediated CD8+ T‐cell suppression via adenosine receptor signaling.^18^ Co‐inhibition of PD‐L1 and CD38 contributed to a stronger anti‐tumor immune response compared with anti‐PD‐L1 monotherapy in a mouse lung cancer model.^18^ Thus, the opportunity remains to develop regimens that incorporate an anti‐CD38 monoclonal antibody as a partner for patients with lymphoma. Cemiplimab (Cemi) is a recombinant human IgG4 monoclonal antibody that binds to PD‐1 and blocks its interaction with PD‐L1 and PD‐L2.^19^ Cemi is approved for treating patients with metastatic or locally advanced cutaneous squamous cell carcinoma, basal cell carcinoma, and non‐small cell lung cancer with high PD‐L1 expression.^19^ Preliminary studies showed that Cemi monotherapy is well tolerated and has minimal clinical activity in patients with HL and B‐cell NHL.^8^

Isatuximab (Isa) is an IgG1 monoclonal antibody that targets a specific epitope on CD38 and kills CD38+ cells from hematological malignancies via multiple mechanisms.^17^, ^20^ Notably, Isa demonstrated potent antitumor activity against diverse CD38+ B‐cell lymphoma, DLBCL, MM, and leukemia cell lines as well as xenograft models derived from these cell lines.^17^ Based on the Phase 3 ICARIA‐MM and IKEMA studies (NCT02990338; NCT03275285),^21^, ^22^ Isa has been approved in combination with pomalidomide and dexamethasone or carfilzomib and dexamethasone, respectively, in MM.

In view of preclinical evidence for the activity of Isa in lymphoma, coupled with early studies showing suboptimal clinical activity of single‐agent anti‐PD‐1 antibodies in HL, DLBCL, and PTCL, we hypothesize that Isa plus Cemi (Isa + Cemi) will have a synergistic effect in patients with lymphoma. Currently, data on Isa activity in PTCL are lacking; however, we included patients with PTCL because CD38, PD‐1, and PD‐L1 are expressed in certain types of PTCL.^2^ In this Phase 1/2 study (NCT03769181), we evaluated the safety, efficacy, and pharmacokinetics of Isa + Cemi in patients with relapsed and refractory cHL, DLBCL, and PTCL. Phase 1 primary objectives were to characterize the safety and tolerability of Isa + Cemi and to confirm the recommended Phase 2 dose (RP2D). In Phase 2, we assessed the CR rate of Isa + Cemi in anti‐PD‐1/PD‐L1 naïve patients with cHL (Cohort A1) and objective response rate (ORR) in anti‐PD‐1/PD‐L1 progressors with cHL (Cohort A2), anti‐PD‐1/PD‐L1 naïve patients with DLBCL (Cohort B), and anti‐PD‐1/PD‐L1 naïve patients with PTCL (Cohort C).

## 2 PATIENTS AND METHODS

### 2.1 Study design and participants

This was a Phase 1/2 multicenter, non‐comparative, open‐label study. Patients were enrolled at 23 study sites in seven countries (France, Italy, Republic of Korea, Netherlands, Portugal, Spain, and Taiwan).

Eligible patients were ≥12 years old and had disease location amenable to tumor biopsy at baseline, measurable disease, and histologically confirmed advanced cHL, DLBCL, or PTCL that had relapsed or progressed after previous therapy. The number of previous therapies required for inclusion differed by cohort: ≥3 (cHL anti‐PD‐1/PD‐L1 naïve), 1 anti–PD‐1/PD‐L1–containing regimen (cHL anti‐PD‐1/PD‐L1 progressors), 2 (DLBCL), and 1 (PTCL). Key exclusion criteria were prior exposure to anti‐CD38, anti–PD‐1/PD‐L1 (except for cHL anti–PD‐1/PD‐L1 progressors), anti‐PD‐L2, anti‐CD137, anti‐CTLA4, or anti‐LAG3 agents, evidence of other immune‐related disease/conditions, Eastern Cooperative Oncology Group (ECOG) performance status ≥2, poor bone marrow reserve, and poor organ function. Among cHL anti‐PD‐1/PD‐L1 progressors, 3 patients were refractory to a prior anti‐PD‐1 agent. One patient who had a partial response (PR) with prior pembrolizumab developed progressive disease upon rechallenge.

**FIGURE 1:**
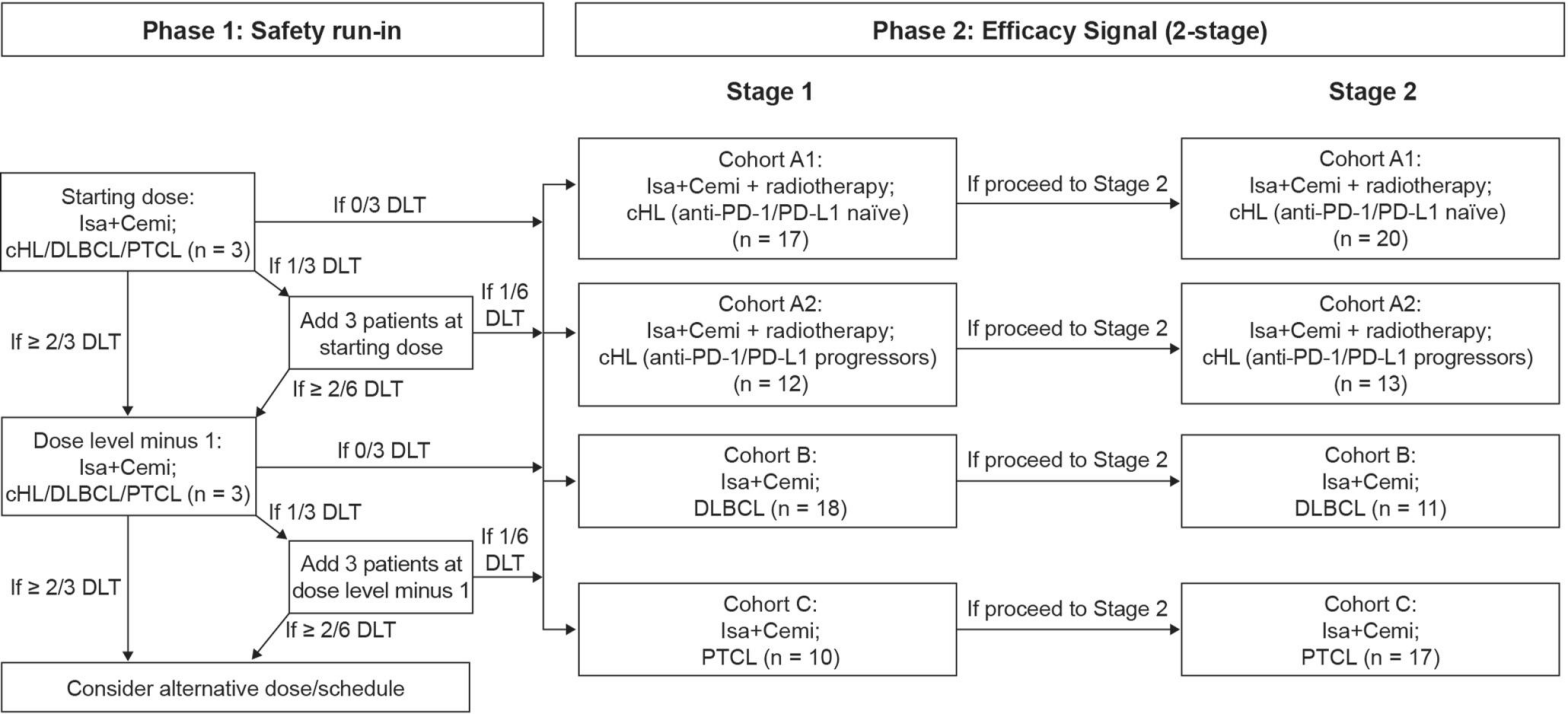
Study design. Cemi, cemiplimab; cHL, classic Hodgkin lymphoma (HL); DLBCL, diffuse large B‐cell lymphoma; DLT, dose‐limiting toxicity; Isa, isatuximab; PD‐1, programmed death‐1; PD‐L1, programmed death ligand‐1; PTCL, peripheral T‐cell lymphoma

The study was in two parts (study design in Figure 1). Eligible patients with cHL, DLBCL, or PTCL were enrolled in Phase 1 and RP2D was determined according to the dose‐limiting toxicities (DLTs) in Cycle 1. Patients were treated with Isa + Cemi at the starting dose; dose de‐escalation to a dose level minus one or an alternative dose/schedule was planned if DLTs occurred in ≥2/3 patients in Cycle 1 (see Procedures for drug doses). Patients were treated in Stage 1 of Phase 2 at the RP2D until disease progression or unacceptable toxicity. Phase 2 included four cohorts: A1, A2, B, and C. Patients treated at the RP2D of Isa + Cemi during Phase 1 were included in the efficacy analysis together with participants of the same indication in Stage 1 of Phase 2. Expected enrollment was 3–12 DLT–evaluable patients in Phase 1 and 37 (Cohort A1), 25 (Cohort A2), 29 (Cohort B), and 27 (Cohort C) patients in Phase 2 (if Stages 1 and 2 were both completed). A prespecified, interim efficacy analysis was planned independently for each cohort. The criteria required to advance a treatment cohort from Phase 2, Stage 1 to Stage 2 of Phase 2 were: CR in 4/17 (23.5%) patients in Cohort A1, and CR or PR in 3/12 (25.0%) patients in Cohort A2, 8/18 (44.4%) patients in Cohort B, and 3/10 (30.0%) patients in Cohort C. The actual number of patients enrolled per cohort was based on the interim analysis results, which was preplanned specifically to avoid treating patients if the combination did not have a synergistic effect.

### 2.2 Procedures

In Phase 1, patients received a starting dose of Isa (10 mg/kg) every week in Cycle 1, every 2 weeks in Cycles 2–6, and every 3 weeks thereafter. Cemi was given at 250 mg every 2 weeks in Cycles 1–6, and at 350 mg every 3 weeks thereafter. The cycle duration was 28 days for Cycles 1–6, then 21 days in subsequent cycles. All participants received the following premedications (or equivalent), 30–60 min before Isa infusion, to prevent or reduce the incidence or severity of infusion reactions: oral acetaminophen 650–1000 mg, ranitidine 50 mg intravenously (IV), diphenhydramine 25–50 mg IV, methylprednisolone 100 mg IV, and oral montelukast 10 mg.

### 2.3 Outcomes

In Phase 1, safety and tolerability were assessed based on DLTs in Cycle 1, treatment‐emergent adverse events (TEAEs) or serious TEAEs, and laboratory abnormalities. TEAEs and laboratory abnormalities were graded using the National Cancer Institute Common Terminology Criteria for Adverse Events version 5.0.

Disease response was determined using positron emission tomography and assessed using the Lugano response criteria, 2014.^23^ For Phase 2, CR rate was defined as the proportion of participants who had complete metabolic response (CMR) as best overall response. Objective response rate was defined as the proportion of participants with CMR or PR as best overall response.

Key secondary endpoints were duration of response (DoR) and progression‐free survival (PFS).

The all‐treated population was the primary analysis population for efficacy and safety analyses and included all participants who received ≥1 Isa or Cemi dose.

### 2.4 Pharmacokinetics

Blood samples were collected at selected time points during Cycle 1 (predose, end of infusion [EOI], EOI+4h, start of infusion [SOI]+72h, SOI+144h) to perform Isa pharmacokinetics by non‐compartmental analysis using Phoenix WinNonlin® version 8.2 (Pharsight). Gyrolab Platform, a quantitative Sandwich immunoassay using biotinylated anti‐Isa antibodies bound by streptavidin beads within the Gyrolab Bioaffy CD microstructure for capture and Alexa Fluor® 647‐conjugated CD38 antibody for detection, was used to measure functional Isa (Isa with ≥1 site available to bind target) plasma levels, with a lower limit of quantitation of 5.0 μg/ml and an upper limit of quantitation of 500 μg/ml.

### 2.5 Statistical analysis

The sample size was determined based on the primary efficacy endpoint. An interim efficacy analysis was performed when the first 18 (Cohort A1), 12 (Cohort A2), 17 (Cohort B), and 11 (Cohort C) patients in Phase 2 had been treated and the last patient in Phase 2 had been followed up for 24 weeks (data cutoff: 5 May 2020, for Cohorts B and C; 18 August 2020, for Cohort A1; 15 February 2021, for Cohort A2).

CR and ORR rates were summarized using descriptive statistics. A 90% two‐sided confidence interval (CI) was computed using the Clopper–Pearson method. Efficacy evaluation was based on Simon's 2‐stage design with 85% power at a 1‐sided alpha level of 5% for each cohort. Duration of response, median PFS, and 95% CIs were calculated using the Kaplan–Meier method. Individual concentrations and pharmacokinetic parameters of Isa were summarized by descriptive statistics.

## 3 RESULTS

### 3.1 Patients

In cohorts A1, A2, B, and C, 18, 12, 17, and 11 patients were enrolled, respectively. Patient baseline characteristics are shown in Table 1. In Phase 1, 6 patients in each cohort were treated with a starting dose of Isa + Cemi to determine the RP2D. No DLTs were observed during Cycle 1; thus, the starting dose was confirmed as the RP2D, obviating dose de‐escalation.

**TABLE 1:** Patient baseline characteristics

	Cohort A1: cHL (anti‐PD‐1/PD‐L1 naïve) (*n* = 18)	Cohort A2: cHL (anti‐PD‐1/PD‐L1 progressors) (*n* = 12)	Cohort B: DLBCL (*n* = 17)	Cohort C: PTCL (*n* = 11)
Age, years, median (range)	36.0 (21–87)	33.0 (19–68)	64.0 (23–75)	69.0 (50–77)
Male	10 (55.6)	7 (58.3)	12 (70.6)	7 (63.6)
ECOG performance status				
0	14 (77.8)	9 (75.0)	5 (29.4)	3 (27.3)
1	4 (22.2)	3 (25.0)	11 (64.7)	8 (72.7)
2	0	0	1 (5.9)	0
Staging				
Stage I	0	0	0	0
Stage II	1 (5.6)	4 (33.3)	1 (5.9)	1 (9.1)
Stage II bulky	1 (5.6)	0	1 (5.9)	0
Stage III	4 (22.2)	4 (33.3)	3 (17.6)	1 (9.1)
Stage IV	12 (66.7)	4 (33.3)	12 (70.6)	9 (81.8)
Number of prior regimens				
1	0	0	0	10 (90.9)
2	0	0	14 (82.4)	0
>2	18 (100)	12 (100)	3 (17.6)	1 (9.1)
Subtype				
Lymphocyte‐rich cHL	1 (5.6)	1 (9.1)	NA	NA
Mixed‐cellularity cHL	3 (16.7)	2 (18.2)	NA	NA
Nodular‐sclerosis cHL	14 (77.8)	8 (72.7)	NA	NA
DLBCL, NOS	NA	NA	12 (70.6)	NA
High‐grade lymphomas with translocations of *MYC* and *BCL2* and/or *BCL6* (double/triple‐hit lymphoma)	NA	NA	1 (5.9)	NA
Other	NA	NA	3 (17.6)	NA
T‐cell/histiocyte‐rich large B‐cell lymphoma	NA	NA	1 (5.9)	NA
Angioimmunoblastic T‐cell lymphoma	NA	NA	NA	5 (45.5)
PTCL, NOS	NA	NA	NA	6 (54.5)
Time from initial diagnosis to first dose in years, median (range)	2.56 (0.8–8.3)	3.38 (1.3–20.9)	1.19 (0.3–7.7)	1.26 (0.3–6.5)
International Prognostic Score at study entry^a^				
0	0	2 (16.7)	0	0
1	4 (22.2)	4 (33.3)	1 (5.9)	1 (9.1)
2	6 (33.3)	3 (25.0)	1 (5.9)	2 (18.2)
3	4 (22.2)	3 (25.0)	10 (58.8)	5 (45.5)
4	1 (5.6)	0	4 (23.5)	3 (27.3)
5	2 (11.1)	0	1 (5.9)	0
6	1 (5.6)	0	0	0

*Note:* All data are shown as *n* (%), except where otherwise indicated.

Abbreviations: *BCL*, B‐cell lymphoma; cHL, classic Hodgkin's lymphoma; DLBCL, diffuse large B‐cell lymphoma; ECOG, Eastern Cooperative Oncology Group; *MYC*, myelocytomatosis; NA, not applicable; NOS, not otherwise specified; PD‐1, programmed death‐1; PD‐L1, programmed death ligand‐1; PTCL, peripheral T‐cell lymphoma.

^a^ Different risk factors are used to calculate the International Prognostic Score for cHL, DLBCL, and PTCL. For cHL cohorts, 1 point is added with the presence of each of the following criteria: age ≥45 years; albumin <4 g/dl; hemoglobin <10.5 g/dl; leukocytosis (white blood cell count ≥15,000/mm^3^); lymphocytopenia (lymphocyte count <8% of white blood cell count, and/or lymphocyte count <600/mm^3^); male; stage IV disease. For DLBCL and PTCL cohorts, 1 point is added with the presence of each of the following criteria: age >60; Ann Arbor stage III or IV; ECOG performance status ≥2; serum lactate dehydrogenase level >1 × normal; >1 extranodal site.

Exposure to Isa + Cemi was longer in the cHL cohorts compared with the DLBCL and PTCL cohorts. Median (range) duration of exposure and number of cycles, respectively were 33.0 (6–69) weeks and 8.5 (1–21) in the cHL anti‐PD‐1/PD‐L1 naïve cohort, 36.1 (4–83) weeks and 10.0 (1–25) in the cHL anti‐PD‐1/PD‐L1 progressors cohort, 4.0 (2–41) weeks and 1.0 (1–11) in the DLBCL cohort, and 4.1 (2–38) weeks and 1.0 (1–10) in the PTCL cohort.

### 3.2 Safety

TEAEs were reported in 83.3% of the patients in the cHL anti‐PD‐1/PD‐L1 naïve cohort and in all patients in the remaining cohorts (Table 2). Grade ≥3 and serious TEAEs, respectively, were more frequent in the DLBCL (70.6% and 58.8%) and PTCL (81.8% and 63.6%) cohorts than in the cHL cohorts (5.6% and 11.1% [cHL anti‐PD‐1/PD‐L1 naïve], 8.3% and 16.7% [cHL anti‐PD‐1/PD‐L1 progressors]). However, treatment‐related any‐grade, Grade ≥3, and serious TEAEs, respectively, were less frequent across all cohorts (50.0%, 0%, 0% [cHL anti‐PD‐1/PD‐L1 naïve]; 91.7%, 0%, 8.3% [cHL anti‐PD‐1/PD‐L1 progressors]; 52.9%, 5.9%, 11.8% [DLBCL]; 72.7%, 27.3%, 18.2% [PTCL]). No patients in the cHL cohorts reported TEAEs leading to definitive discontinuation, compared with 5.9% in the DLBCL cohort and 27.3% in the PTCL cohort (Table 2).

**TABLE 2:** Safety summary by cohort, all‐treated population

	Cohort A1: cHL (anti‐PD‐1/PD‐L1 naïve) (*n* = 18)	Cohort A2: cHL (anti‐PD‐1/PD‐L1 progressors) (*n* = 12)	Cohort B: DLBCL (*n* = 17)	Cohort C: PTCL (*n* = 11)
Any TEAE	15 (83.3)	12 (100)	17 (100)	11 (100)
Grade ≥3 TEAE	1 (5.6)	1 (8.3)	12 (70.6)	9 (81.8)
Grade 5 TEAE (fatal outcome)	0	0	4 (23.5)	2 (18.2)
SAEs	2 (11.1)	2 (16.7)	10 (58.8)	7 (63.6)
Treatment‐related^a^ TEAEs	9 (50.0)	11 (91.7)	9 (52.9)	8 (72.7)
Treatment‐related^a^ Grade ≥3 TEAE	0	0	1 (5.9)	3 (27.3)
Treatment‐related SAE	0	1 (8.3)	2 (11.8)	2 (18.2)
TEAE leading to treatment discontinuation	0	0	1 (5.9)	3 (27.3)
Any AESI^b^	7 (38.9)	8 (66.7)	7 (41.2)	7 (63.6)
Any IR	7 (38.9)	9 (75.0)	8 (47.1)	8 (72.7)

*Note:* All data are shown as *n* (%).

Abbreviations: AE, adverse event; AESI, adverse event of special interest; cHL, classic Hodgkin's lymphoma; DLT, dose‐limiting toxicity; IR, infusion reaction; PD‐1, programmed death‐1; PD‐L1, programmed death ligand‐1; PI3K, phosphoinositide 3‐kinase; PTCL, peripheral T‐cell lymphoma; SAE, serious adverse event; TEAE, treatment‐emergent adverse event.

^a^ Treatment‐related TEAEs are TEAEs related to at least one drug of the combination.

^b^ AESIs include Grade ≥2 IRs, Grade ≥3 immune‐related TEAEs, immune‐related AEs of any grade in a patient previously treated with a PI3K inhibitor (only applicable for patients who received cemiplimab), DLTs as defined in Phase 1, pregnancy, symptomatic overdose with investigational medicinal product/non‐investigational medicinal product.

No Grade 5 (fatal) TEAEs were reported in the cHL cohorts. There were 4 deaths reported during the on‐treatment period in the DLBCL cohort (2 progressive disease, 1 intestinal perforation, 1 urinary tract infection) and 2 deaths in the PTCL cohort (1 unknown cause, 1 progressive disease); however, no deaths were treatment‐related (Table 2).

TEAEs reported in ≥20% of patients are shown in Table 3. Most TEAEs were Grade 1/2. Infusion‐related reactions were the most common, with an incidence of 38.9%, 75.0%, 52.9%, and 72.7% in cHL anti‐PD‐1/PD‐L1 naïve, cHL anti‐PD‐1/PD‐L1 progressor, DLBCL, and PTCL cohorts, respectively. Among Grade ≥3 TEAEs, infusion‐related reactions and pyrexia were reported in 1 (9.1%) patient each in the PTCL cohort, peripheral edema and abdominal pain in 1 (5.9%) patient each, and decreased appetite in 2 (11.8%) patients in the DLBCL cohort. Other commonly reported TEAEs were pyrexia (22.2%) in the cHL anti‐PD‐1/PD‐L1 naïve cohort; nausea (33.3%), pyrexia (25.0%), diarrhea (25.0%), and pruritus (25.0%) in the cHL anti‐PD‐1/PD‐L1 progressors cohort; and abdominal pain (29.4%), peripheral edema (29.4%), decreased appetite (23.5%), diarrhea (23.5%), fatigue (23.5%), and nausea (23.5%) in the DLBCL cohort.

**TABLE 3:** TEAEs in ≥20% of patients by cohort

	Cohort A1: cHL (anti‐PD‐1/PD‐L1 naïve) (*n* = 18)		Cohort A2: cHL (anti‐PD‐1/PD‐L1 progressors) (*n* = 12)		Cohort B: DLBCL (*n* = 17)		Cohort C: PTCL (*n* = 11)	
	All grades	Grade ≥3	All grades	Grade ≥3	All grades	Grade ≥3	All grades	Grade ≥3
Any event	15 (83.3)	1 (5.6)	12 (100)	1 (8.3)	17 (100)	12 (70.6)	11 (100)	9 (81.8)
Infusion‐related reaction	7 (38.9)	0	9 (75.0)	0	9 (52.9)	0	8 (72.7)	1 (9.1)
Pyrexia	4 (22.2)	0	3 (25.0)	0	2 (11.8)	0	2 (18.2)	1 (9.1)
Diarrhea	3 (16.7)	0	3 (25.0)	0	4 (23.5)	0	1 (9.1)	0
Peripheral edema	3 (16.7)	0	0	0	5 (29.4)	1 (5.9)	1 (9.1)	0
Pruritus	2 (11.1)	0	3 (25.0)	0	0	0	1 (9.1)	0
Abdominal pain	2 (11.1)	0	1 (8.3)	0	5 (29.4)	1 (5.9)	0	0
Decreased appetite	2 (11.1)	0	0	0	4 (23.5)	2 (11.8)	0	0
Nausea	1 (5.6)	0	4 (33.3)	0	4 (23.5)	0	0	0
Fatigue	0	0	1 (8.3)	0	4 (23.5)	0	1 (9.1)	0
Hematological laboratory abnormalities								
Anemia	16 (88.9)	1 (5.6)	7 (58.3)	0	16 (100)	2 (12.5)	11 (100)	2 (18.2)
Lymphopenia	12 (66.7)	5 (27.8)	3 (25.0)	1 (8.3)	14 (87.5)	7 (43.8)	10 (90.9)	4 (36.4)
Neutropenia	3 (16.7)	0	–	–	6 (37.5)	3 (18.8)	6 (54.5)	2 (18.2)
Leukopenia	10 (55.6)	0	2 (16.7)	0	13 (81.3)	3 (18.8)	8 (72.7)	2 (18.2)
Thrombocytopenia	7 (38.9)	0	3 (25.0)	0	11 (68.8)	2 (12.5)	8 (72.7)	4 (36.4)

*Note:* All data are shown as *n* (%).

Abbreviations: cHL, classic Hodgkin's lymphoma; DLBCL, diffuse large B‐cell lymphoma; PD‐1, programmed death‐1; PD‐L1, programmed death ligand‐1; PTCL, peripheral T‐cell lymphoma; TEAE, treatment‐emergent adverse event.

The most commonly reported all‐grade hematological laboratory abnormality was anemia (88.9% [cHL anti‐PD‐1/PD‐L1 naïve], 58.3% [cHL anti‐PD‐1/PD‐L1 progressors], 100% [DLBCL and PTCL]). The most frequent Grade ≥3 hematological laboratory abnormalities were lymphopenia in all cohorts (27.8% [cHL anti‐PD‐1/PD‐L1 naïve], 8.3% [cHL anti‐PD‐1/PD‐L1 progressors], 43.8% [DLBCL], and 36.4% [PTCL]), and thrombocytopenia (36.4%) in the PTCL cohort (Table 3).

### 3.3 Efficacy

Among the all‐treated population, response rate was 55.6%, 33.3%, 5.9%, and 9.1% in cHL anti‐PD‐1/PD‐L1 naïve, cHL anti‐PD‐1/PD‐L1 progressor, DLBCL, and PTCL cohorts, respectively (Table 4). Complete metabolic response was observed in 5 (27.8%) cHL anti‐PD‐1/PD‐L1 naïve patients, 2 (16.7%) cHL anti‐PD‐1/PD‐L1 progressors, and 1 (5.9%) DLBCL patient. No PTCL patients achieved CMR. Among the 10 responders in the cHL anti‐PD‐1/PD‐L1 naïve cohort, five experienced radiological progression or death with a median (range) DoR of 5.79 (1.41–9.3) months; 2/4 responders in the cHL anti‐PD‐1/PD‐L1 progressors cohort had a DoR of 3.19 and 7.56 months.

**TABLE 4:** Summary of response rate per Lugano 2014 criteria, all‐treated population

	Cohort A1: cHL (anti‐PD‐1/PD‐L1 naïve) (*n* = 18)	Cohort A2: cHL (anti‐PD‐1/PD‐L1 progressors) (*n* = 12)	Cohort B: DLBCL (*n* = 17)	Cohort C: PTCL (*n* = 11)
Overall response				
Responders (CR or PR)	10 (55.6)	4 (33.3)	1 (5.9)	1 (9.1)
Best overall response				
Complete metabolic response/complete response	5 (27.8)	2 (16.7)	1 (5.9)	0
Partial metabolic response/partial response	5 (27.8)	2 (16.7)	0	1 (9.1)
No metabolic response/stable disease	1 (5.6)	3 (25.0)	0	1 (9.1)
Progressive metabolic disease/progressive disease	6 (33.3)	5 (41.7)	7 (41.2)	4 (36.4)
Not evaluable^a^	1 (5.6)	0	9 (52.9)	5 (45.5)

*Note:* All data are shown as *n* (%).

Abbreviations: cHL, classic Hodgkin's lymphoma; CR, complete response; DLBCL, diffuse large B‐cell lymphoma; PD‐1, programmed death‐1; PD‐L1, programmed death ligand‐1; PR, partial response; PTCL, peripheral T‐cell lymphoma.

^a^ Including patients with no post‐baseline evaluation prior to the initiation of a new anti‐cancer therapy or the data cutoff date.

**FIGURE 2:**
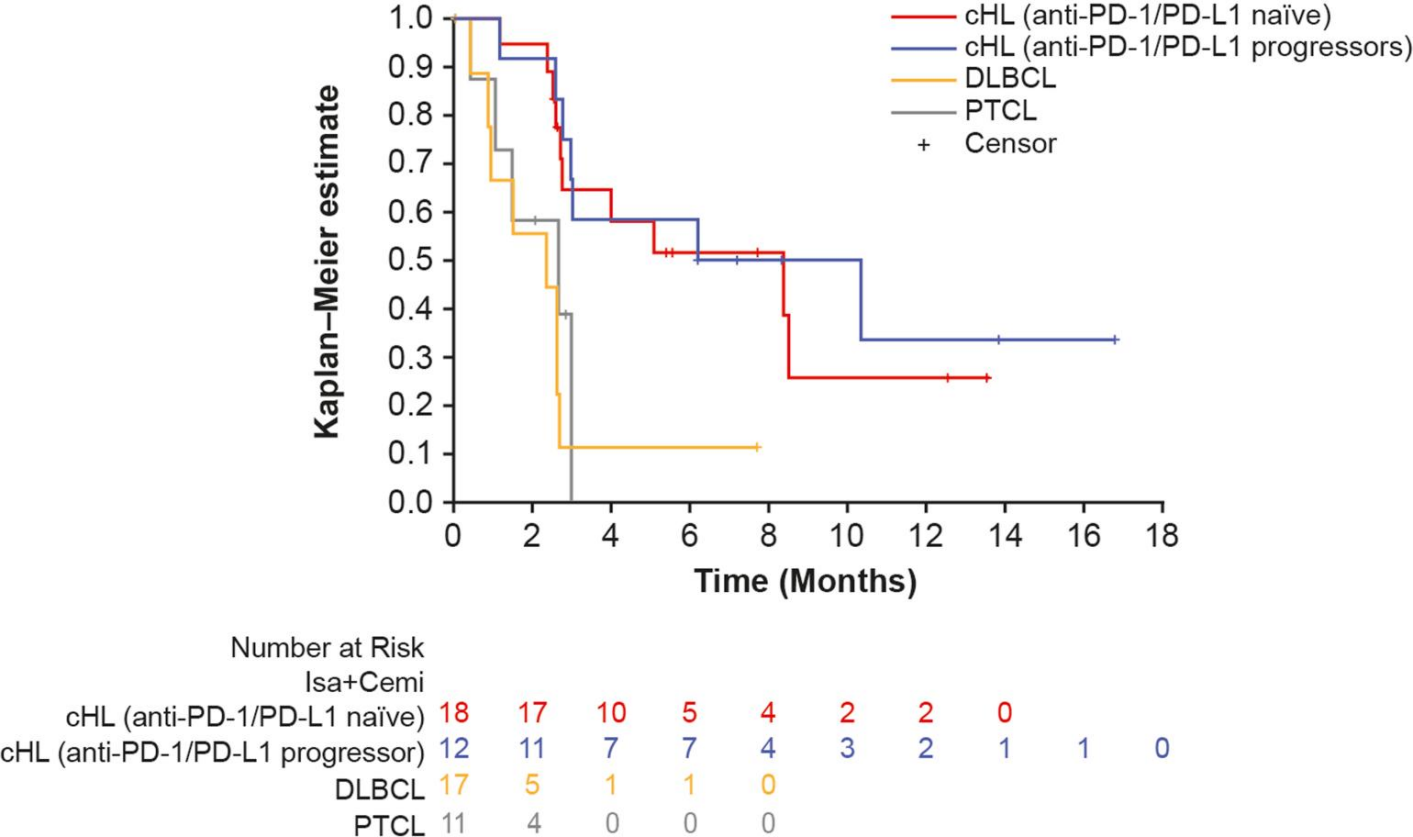
Progression‐free survival (PFS): Kaplan–Meier estimates by cohort, all‐treated population. Cemi, cemiplimab; cHL, classic Hodgkin lymphoma (HL); DLBCL, diffuse large B‐cell lymphoma; PD‐1, programmed death‐1; PD‐L1, programmed death ligand‐1; PTCL, peripheral T‐cell lymphoma; Isa, isatuximab

Median PFS (95% CI) was 8.38 months (2.73–not calculable [NC]), 8.28 months (2.60–NC), 2.37 months (0.46–2.69), and 2.66 months (0.43–2.99) in cHL anti‐PD‐1/PD‐L1 naïve, cHL anti‐PD‐1/PD‐L1 progressors, DLBCL, and PTCL cohorts, respectively (Figure 2).

### 3.4 Pharmacokinetics

Mean Isa maximum plasma concentration (*C*_max_) and area under the concentration versus time curve over 1 week (AUC_1 week_) were, respectively, 226 μg/ml and 22,500 μg.h/ml in the cHL anti‐PD‐1/PD‐L1 naïve cohort, 265 μg/ml and 26,600 μg.h/ml in the cHL anti‐PD‐1/PD‐L1 progressors cohort, 253 μg/ml and 23,700 μg.h/ml in the DLBCL cohort, and 181 μg/ml and 15,000 μg.h/ml in the PTCL cohort (Table 5). Pharmacokinetic analyses suggested no effect of Cemi on Isa exposure (*C*_max_, AUC_1week_).

**TABLE 5:** Isa pharmacokinetic parameters

Mean ± SD (Geometric mean) [CV%]	Cohort A1: cHL (anti‐PD‐1/PD‐L1 naïve) (*n* = 17)	Cohort A2: cHL (anti‐PD‐1/PD‐L1 progressors) (*n* = 12)	Cohort B: DLBCL (*n* = 14)	Cohort C: PTCL (*n* = 7)	All (*N* = 50)
*C*_max_	226 ± 48.1	265 ± 38.8	253 ± 29.1	181 ± 35.7	237 ± 47.1
(μg/ml)	(221) [21]	(262) [15]	(251) [12]	(178) [20]	(232) [20]
*t*_max_‎^a^	5.95	5.23	5.26	4.57	5.17
(h) ^b^	(2.00–8.92)	(3.00–8.83)	(2.60–10.5)	(2.33–7.45)	(2.00–10.5)
AUC_1 week_	22,500 ± 5770	26,600 ± 4030	23,700 ± 4960	15,000 ± 4680	22,700 ± 6030
(μg•h/mL)	(21,800) [26]	(26,300) [15]^c^	(23,200) [21]	(14,300) [31]	(21,800) [27]^d^

^a^ Median.

^b^ Min–max.

^c^ *n* = 11.

^d^ *n* = 49.

Abbreviations: AUC, area under curve; cHL, classic Hodgkin's lymphoma; *C*_max_, maximum concentration; CV, coefficient of variation; DLBCL, diffuse large B‐cell lymphoma; Isa, isatuximab; PD‐1, programmed death‐1; PD‐L1, programmed death ligand‐1; PTCL, peripheral T‐cell lymphoma; SD, standard deviation; *t*_max_, time to reach *C*_max_.

## 4 DISCUSSION

In patients with relapsed/refractory lymphoma, treatment beyond the first line remains challenging and represents a significant unmet need.^24^, ^25^, ^26^ Monoclonal antibodies that block the PD‐1/PD‐L1 axis have demonstrated limited clinical benefit in lymphoma. Although nivolumab and pembrolizumab are approved for relapsed/refractory cHL, only a small subset (around 20%–28%) of the patients achieved complete remission.^3^, ^4^ Similar outcomes (12% CR) were reported with Cemi monotherapy in an early‐phase study in patients with HL.^8^ Combining nivolumab with brentuximab vedotin (anti‐CD30 antibody–drug conjugate) increased CR rate to 61% in the interim analysis of a Phase 1/2 study of relapsed/refractory patients with cHL.^27^ In a Phase 1b study with nivolumab monotherapy, ORR was achieved in 4/11 (36%) patients with DLBCL and 2/5 (40%) patients with PTCL.^7^ Among patients with DLBCL treated with Cemi monotherapy, ORR was achieved in 3/18 (17%) patients.^8^ Although the reason for suboptimal efficacy of these agents in lymphoma is unclear, their promising anti‐tumor activity in a subset of lymphomas has spurred interest in the development of newer, potentially more effective checkpoint inhibitors and combinations of agents with different mechanisms of action for the treatment of patients with lymphoma.^1^

The expression of CD38 in lymphoma, together with preclinical evidence for anti‐CD38 antibodies against CD38+ lymphoma, suggests potential utility for these agents in patients with CD38+ lymphoma. The anti‐CD38 antibodies, Isa, daratumumab, and MOR202 kill CD38+ lymphoma cells via antibody‐dependent cellular cytotoxicity and antibody‐dependent cellular phagocytosis^9^, ^16^, ^17^ In addition, Isa has strong anti‐apoptotic activity in lymphoma cells in the absence of crosslinking agents.^17^

Several studies suggest a role for CD38 in the immunomodulatory and pro‐tumoral microenvironment. CD38 is expressed in many immunosuppressive cell types^9^, ^28^, ^29^, ^30^, ^31^; targeting CD38‐expressing immune cells triggers anti‐tumor immunomodulatory mechanisms.^17^, ^32^, ^33^, ^34^, ^35^, ^36^ CD38 is upregulated in mouse models of lung cancer and melanoma that have acquired resistance to anti‐PD‐1/anti‐PD‐L1 blockade, and correlates with CD8+ T‐cell impairment.^28^, ^29^, ^37^ Concurrent inhibition of CD38 and PD‐L1 in these murine models has been shown to substantially reduce primary tumor burden and metastases.^18^

To address whether co‐inhibition of PD‐1 and CD38 may benefit patients with lymphoma, we assessed the safety, efficacy, and pharmacokinetics of Isa + Cemi in patients with cHL, DLBCL, and PTCL. During the Phase 1 safety run‐in part of this study, no DLTs were observed during Cycle 1 and the RP2D was confirmed for Phase 2. Isa + Cemi had a manageable safety profile with no new safety signals compared with known effects of the individual drugs. Infusion‐related reactions were the most common events. Grade ≥3, Grade 5, and serious TEAEs were higher in the DLBCL and PTCL cohorts compared with the cHL cohorts. Treatment‐related TEAEs were less frequent in all cohorts.

Modest clinical efficacy was observed in patients with cHL, with responses achieved in patients who had or had not previously received anti‐PD‐1/PD‐L1 therapy. However, the study was terminated in view of other more efficacious therapeutic options available for this patient population. For the DLBCL and PTCL cohorts, results of the interim efficacy analysis did not meet prespecified criteria (i.e., ≥8/17 and ≥3/11 responders in the DLBCL and PTCL cohorts, respectively) to continue enrollment in Stage 2 of Phase 2. Consequently, accrual was stopped for the DLBCL and PTCL cohorts.

Most patients with DLBCL were primary refractory/bulky and discontinued within weeks from treatment initiation; in such patients, a more aggressive combination therapy may be needed. Overall, these results demonstrated that the combination was not very active in DLBCL and PTCL populations. This is consistent with limited clinical benefit of daratumumab monotherapy in Phase 2 studies with patients with relapsed/refractory B‐cell NHL subtypes and natural killer/T‐cell lymphoma.^38^, ^39^ The clinical development of next‐generation anti‐CD38 antibodies, as well as new modalities targeting this tumor antigen, may provide more effective treatment options for improved outcomes in the advanced lymphoid malignancy space.

Pharmacokinetic parameters of Isa in the current study were consistent with those observed in a recent Phase 1/2 study of Isa + Cemi in patients with metastatic castration‐resistant prostate cancer or advanced non‐small cell lung cancer, where the same assay method was used, suggesting no effect of Cemi on Isa exposure (*C*_max_, AUC_1week_).^40^ Similar to the current study in lymphoma, this study demonstrated a lack of efficacy of Isa + Cemi in small patient cohorts with these advanced malignancies.

## 5 CONCLUSION

Isa + Cemi had a manageable safety profile. Limited clinical efficacy was observed in patients with cHL who had prior anti‐PD‐1/PD‐L1 exposure, as well as in those who did not. However, we did not observe a synergistic effect of Isa in combination with Cemi in patients with cHL, DLBCL, or PTCL.

## AUTHOR CONTRIBUTIONS

Rao Saleem was responsible for study oversight. Carmelo Carlo‐Stella, Pier Luigi Zinzani, Anna Sureda, Luis Araújo, Olivier Casasnovas, Cecilia Carpio, Su‐Peng Yeh, Krimo Bouabdallah, Guillaume Cartron, Won Seog Kim, Raul Cordoba, Youngil Koh, Alessandro Re, Daniela Alves, Martine Chamuleau, Steven Le Gouill, Armando López‐Guillermo, Ilídia Moreira, Marjolein W. M. van der Poel, and Vincent Ribrag were investigators in the study and contributed to data acquisition and analysis. Vincent Ribrag designed the overall study and for DLBCL, Carmelo Carlo‐Stella for cHL, and Pier Luigi Zinzani for PTCL. Giovanni Abbadessa, Robin Meng, Ran Ji, Lucie Lépine, and Rao Saleem contributed to analysis and interpretation of data for the work. All authors revised the work for important intellectual content and assume responsibility for data integrity and the decision to submit this manuscript for publication, had full access to the study data, edited and reviewed manuscript drafts, and approved the final version for submission.

## CONFLICT OF INTEREST

CC‐S: *Advisory Role* – Sanofi; *Consultancy* – Sanofi. PLZ: *Honoraria* – Gilead, Incyte, Merck, Novartis, Roche, Sanofi, Takeda. AS: *Consultancy* –Bluebird, BMS, Genmab, Janssen, Kite, MSD, Novartis, Roche, Takeda; *Honoraria* – BMS, Janssen, Kite, MSD, Novartis, Roche, Sanofi, Takeda. LA: Nothing to disclose. OC: *Advisory Role* – Gilead/Kite, Roche, Takeda; *Consultancy* – AbbVie, BMS, Gilead/Kite, Janssen, MSD, Roche, Takeda; *Honoraria* – Gilead/Kite, Roche; *Research* *Funding* – Gilead/Kite, Roche, Takeda. CC: *Consultancy* – Regeneron, Takeda; *Honoraria* – BMS, Novartis, Takeda. S‐PY: *Advisory Role* – AbbVie, Amgen, Astellas, Astex, Janssen, Novartis, Sanofi, Takeda; *Honoraria* – AbbVie, Amgen, Astellas, BMS, Janssen, AstraZeneca, Novartis, Roche, Sanofi, Takeda. KB: Nothing to disclose. GC: *Consultancy* – Celgene, Roche; *Honoraria* – AbbVie, Celgene, Gilead, Janssen, Novartis, Roche, Sanofi, Takeda. WSK: *Research* *Funding* – Celltrion, Kyowa Kirin, Pfizer, Roche, Sanofi. RC: *Advisory Role* – AbbVie, AstraZeneca, BeiGene, BMS, Incyte, Janssen, Kite, Kyowa Kirin, Lilly, Roche, Takeda; *Consultancy* – AbbVie, AstraZeneca, BeiGene, BMS, Incyte, Janssen, Kite, Kyowa Kirin, Lilly, Roche, Takeda; *Honoraria* – AbbVie, AstraZeneca, BMS, Janssen, Kite, Roche, Takeda; *Research* *Funding* ‐ Pfizer. YK: Nothing to disclose. AR: Nothing to disclose. DA: *Advisory Role* – AbbVie, Roche; *Consultancy* – AbbVie, AstraZeneca, Gilead, Janssen, Roche, Takeda; *Honoraria* – AbbVie, AstraZeneca, Gilead, Janssen, Roche, Takeda. MC: *Advisory Role* – Novartis; *Research* *Funding* – Celgene, Genmab, GiIead. SLG: *Consultancy* – Roche; *Honoraria* – Roche. AL‐G: *Consultancy* –Celgene/BMS, Gilead/Kite, Incyte, Janssen, Kern Pharma, Pfizer, Roche, Takeda; *Honoraria* – Roche; *Research* *Funding* – Celgene/BMS, Janssen, Roche. IM: Nothing to disclose. MWMvdP: Nothing to disclose. GA, RM, RJ, LL, and RS are employed by Sanofi and may hold stock and/or stock options in the company. VR: *Advisory Role* – AstraZeneca; *Consultancy* – AstraZeneca, Gilead, Incyte, NanoString, Roche; *Honoraria* – AstraZeneca; *Research* *Funding* – argenx, Astex Pharmaceuticals, GSK.

### PEER REVIEW

The peer review history for this article is available at https://publons.com/publon/10.1002/hon.3089.

## ETHICS STATEMENT

The study was conducted in accordance with the Declaration of Helsinki, Council for International Organizations of Medical Sciences International Ethical Guidelines, and the International Conference on Harmonization Good Clinical Practice Guidelines. The relevant independent ethics committees and institutional review boards approved the study protocol. All participants provided written informed consent prior to study enrollment. Clinical trial registration: ClinicalTrials.gov, NCT03769181.

## Data Availability

Qualified researchers can request access to patient‐level data and related study documents including the clinical study report, study protocol with any amendments, blank case report forms, statistical analysis plan, and dataset specifications. Patient‐level data will be anonymized, and study documents will be redacted to protect the privacy of trial participants. Further details on Sanofi's data‐sharing criteria, eligible studies, and process for requesting access are at: https://www.vivli.org.
